# Growth monitoring and promotion service utilization and its associated factors among children less than two years in Ethiopia: A systematic review and meta-analysis

**DOI:** 10.1371/journal.pone.0311531

**Published:** 2024-11-19

**Authors:** Mulat Belay Simegn, Werkneh Melkie Tilahun, Elyas Melaku Mazengia, Aysheshim Belaineh Haimanot, Anteneh Lamesgen Mneneh, Muluye Gebrie Mengie, Bekalu Endalew, Molla Yigzaw Birhanu, Tigabu Kidie Tesfie, Lakew Asmare, Habtamu Geremew

**Affiliations:** 1 Department of Public Health, College of Medicine and Health Sciences, Debre Markos University, Debre Markos, Ethiopia; 2 Department of Epidemiology and Biostatistics, Institute of Public Health, College of Medicine and Health Sciences, University of Gondar, Gondar, Ethiopia; 3 Department of Epidemiology and Biostatistics, School of Public Health, College of Medicine and Health Sciences, Wollo University, Dessie, Ethiopia; 4 College of Health Science, Oda Bultum University, Chiro, Ethiopia; Debre Berhan University, ETHIOPIA

## Abstract

**Introduction:**

Growth monitoring and promotion services are strategies to promote child health and reduce child mortality. Even though Ethiopia is attempting different strategies to cope with the low rate of GMP utilization, the problem is still unresolved.

**Objective:**

Determine the pooled proportion of GMP utilization and its contributing factors among children less than two years in Ethiopia.

**Method:**

The review protocol was registered with PROSPERO, number CRD42023472746. The PRISMA-2020 statement guided the conduct of this review. Electronic databases and grey literature were used. Heterogeneity was evaluated using I^2^. Subgroup analysis was conducted. The random effect model was used to summarize the pooled effect sizes with their respective 95% CI with STATA version 17. To test the small study effect, the funnel plot and Egger’s test were applied.

**Result:**

A total of seven (7) studies with 4027 participants were considered in this meta-analysis. The pooled proportion of GMP utilization reported by seven studies was 25.71% (95%CI: 24.39, 27.04). ANC follow-up (AOR = 2.11; 95% CI: 1.47, 2.76), PNC follow-up (AOR = 1.96; 95% CI: 1.44, 2.49), counseling (AOR = 2.88; 95% CI: 2.09, 3.68), maternal education (AOR = 2.89; 95% CI: 1.66, 4.13), paternal education (AOR = 3.78; 95% CI: 2.25, 5.32), family health card (AOR = 2.31; 95% CI: 1.67, 2.96), and mothers good knowledge towards GMP (AOR = 2.90; 95% CI: 1.72, 4.07) variables were positively associated with GMP service utilization.

**Conclusion and recommendation:**

The pooled proportion of GMP remains low in Ethiopia. ANC and PNC follow-up, counseling, maternal and paternal education, family health cards, maternal knowledge towards GMP were significantly associated. Findings are essential for evidence-based policy making, intervention, and input for ongoing research.

## Introduction

Malnutrition is one of the global public health problems that is impeding development, having terrible effects on people, and contributing to about half of the deaths of children under five [1]. As a global report indicates, 20 million babies are born with low birth weight, 38.3 million are overweight, and 50.5 million are wasted each year [2]. Under nutrition also kills about 10.5 million children worldwide each year; 98% of these deaths are recorded in underdeveloped nations [3]. Ethiopia had a decrease in the number of undernourished children, but still had 38% stunted, 24% underweight, and 10% wasted under five children [4]. The high burden of child malnutrition in developing countries has a huge impact on child growth and development [5]. Weight is a sensitive measurement used to assess growth [6, 7]. Growth monitoring (GM) aims to enhance a child’s nutritional status through routine growth measurement, graphing, and interpretation, as well as appropriate intervention in cases of aberrant development observed. The preventive strategy called growth monitoring and promotion (GMP) monitors, measures, interprets, and analyzes potential causes of either adequate or insufficient child growth. Additionally, it promotes contact and communication, prompts appropriate action, enhances the child’s nutritional condition, and lowers childhood mortality and morbidity [8, 9]. Thus, there is increasing recognition that early life strategies such as growth monitoring and promotion should be taken into account to tackle malnutrition and mortality [10]. The main potential benefits of growth monitoring and promotion services are improvements in nutritional status, enhanced healthcare service utilization, and a consequent decrease in mortality [10, 11]. According to different primary studies reports, parental intention for GMP, misunderstanding of the chart by health workers, and poor intention of mothers for the service [8, 12, 13], antenatal care and postnatal care follow-up [9, 14, 15], maternal and paternal education status [9, 15–18], wealth index[9, 14, 16], maternal knowledge and attitude towards GMP service utilization [15, 16, 18], distance near to the health facility [14–16], use family health card(FHC) [9, 14, 19] and maternal counseling towards GMP [15, 18, 19] were factors associated with growth monitoring and promotion service utilization. The association between the introduction of GMP programs and ensuing improvements to care practices has not been extensively studied [8, 20].

Through health extension initiatives and all primary health care units, the Ethiopian government has been providing monthly growth monitoring and promotion services. This has been made possible by the implementation of the Sequota declaration and the health sector transformation plan, which prioritize early disease detection and treatment as well as nutritional counseling [9, 21–23]. Despite the fact that Ethiopia has made different strategies (comprehensive child care, National Nutritional Strategy, and National Nutrition Program Ⅱ) to increase GMP utilization, It remains low in health facilities as well as at the at the community level [12, 22] due to the lack of participation of caregivers and a poor understanding of the concept of growth monitoring. Additionally, lack of support from health workers, poor referral systems and monitoring, and suboptimal supervision practices, poor implementation were and lack of concrete evidence on the area were the most notified reasons for the low GMP service utilization. There were no any pooled estimate of GMP is conducted yet in Ethiopia. The review’s findings of this study would spark a discussion among stakeholders by providing evidence-based intervention for policy makers, and base for the ongoing public health research. Therefore, this study has addressed a research question, what was the status of pooled estimate on growth monitoring and promotion service utilization and factors associated with it?

## Method and materials

Our systematic reviews and meta-analyses were conducted in accordance with the Preferred Reporting Items for Systematic Reviews and Meta-Analyses, 2020 (PRISMA-2020) reporting checklist [24]. The review protocol has been registered on PROSPERO with an ID of CRD42023472746 (https://www.crd.york.ac.uk/prospero/). The database was searched for the same systematic review to avoid duplications on Embase, PubMed, Epistemonikos, The website (http://www.library.UCSF.edu), PROSPERO, and Cochrane/Wiley library were explored to confirm whether previous systematic reviews or meta-analyses existed. The review question were constructed with CoCoPop(condition, context and, population) for prevalence review.

### Review question

1. What is the pooled GMP service utilization among children’s less than two years old in Ethiopia? and
2. What are the factors associated with GMP service utilization among children’s less than two years old in Ethiopia?

### Eligibility criteria

To define the eligibility criteria, the authors consider CoCoPop (condition, context and population) framework for prevalence review. As we have used CoCoPop mnemonics to construct a clear and meaningful review objective or question: Condition: Growth monitoring and promotion, Context: Ethiopia, Population/Participants: less than two years children.

#### Inclusion criteria’s

The review included articles in Ethiopia, from the first record until December 30/2023 from both electronic database and grey literature source including different Ethiopian universities repository conducted in rural and urban, community- and institutional-based, published in English in each study’s cross-sectional, cohort, and case-control study design.

#### Exclusion criteria

Qualitative studies, reviews, and papers published other than in English, letters, commentaries, editorials, expert opinion studies, and studies duplicated were excluded before analysis.

### Search strategy and information source

All studies were systematically searched through electronic peer-reviewed literature starting from PubMed data-bases and proceed for the rest data-base (Cochrane Library, Scopus, Embase, Epistemonikos, Science Direct), and other grey literature like, Google Scholar, Google, and Ethiopian university repositories by considering CoCoPoP (condition: growth monitoring and promotion, context: Ethiopia, population: children less than two years). Furthermore, a search was conducted through the reference list of all identified articles to uncover additional relevant studies. The study articles were searched from November 01, 2023 to December 30, 2023. The authors were contacted for the articles with incomplete reported data [15]. The systematic searching strategy was conducted using the combination of the following keywords and Boolean operators in different electronic data-base **(Table 1)**.

**Table 1:** Search result for growth monitoring and promotion service utilization among less than two years old children in Ethiopia.

Mesh Heading	Entry terms	Combination	Number of articles	Last searching date
		PubMed		
Growth monitoring and promotion	Growth monitoring, Growth monitoring practice, GMP	(("Growth monitoring and promotion"[Title/Abstract] OR "growth monitoring"[Title/Abstract] OR "Growth monitoring practice"[Title/Abstract] OR GMP[Title/Abstract]) AND ("Less than two years"[Title/Abstract] OR "Under two years"[Title/Abstract] OR Infant*[Title/Abstract] OR Childs[Title/Abstract] OR Children[Title/Abstract] OR "0–23 months"[Title/Abstract])) AND (Ethiopia[Title/Abstract] OR "Ethiopia Federal Democratic Republic"[Title/Abstract] OR EFDR[Title/Abstract] OR Ethiopian[Title/Abstract] OR "Region of Ethiopia"[Title/Abstract])	15	30/12/2023
Ethiopia	Federal Democratic Republic of Ethiopia, Ethiopian, Regions of Ethiopia, FDRE			
Less than two years children	Under two years, 0–23 month children, Infant, Infants, children, Childs			
**Embase**				
		(“Growth monitoring and promotion”/exp OR “Growth monitoring” OR “Growth monitoring practice”/exp OR GMP) AND (“Less than two years”:ti,ab,kw OR “Under two years”:ti,ab,kw OR “0–23 month”:ti,ab,kw OR Infant OR Children:ti,ab,kw OR Childs:ti,ab,kw OR Infants:ti,ab,kw) AND Ethiopia:ti,ab,kw	29	
**SCOPUS**				
		(TITLE-ABS-KEY("Growth monitoring and promotion" OR "Growth monitoring " OR "Growth monitoring practice" OR GMP) AND TITLE-ABS-KEY("Less than two years" OR "under two years" OR Children OR "Childs" OR Infants) AND TITLE-ABS-KEY(Ethiopia))	34	30/12/2023
**SCIENCEDIRECT**				
		(("Growth monitoring and promotion" OR "Growth monitoring" OR “Growth monitoring practice” OR GMP) AND ("Less than two years" OR “Under two years” OR Infants OR Children OR Childs“0–23 months”) AND Ethiopia))	20	30/12/2023
**Epistemonikos**				
		(title: ("Growth monitoring and promotion" OR "Growth monitoring" OR "Growth monitoring practice" OR GMP)) OR abstract:("Growth monitoring and promotion" OR "Growth monitoring" OR "Growth monitoring practice" OR GMP))) AND (title:("Less than two years" OR "Under two years" OR Infants OR Infant OR Childs OR Children "0–23 months") OR abstract:("Less than two years" OR "Under two years" OR Infants OR Infant OR Childs OR Children OR "0–23 months")) AND (title:(Ethiopia) OR abstract:(Ethiopia))	14	
**Cochrane Library**				
		“Growth monitoring and promotion” OR “growth monitoring” "Growth monitoring practice" OR GMP in Title Abstract Keyword AND "Less than two years" OR “Under two years” OR Infants OR Infant OR Childs OR Children OR "0–23 months" in Title Abstract Keyword AND Ethiopia OR "Ethiopia Federal Democratic Republic of Ethiopia" OR FDRE	25	
**Google Scholar**				
		((“Growth monitoring and promotion” OR “growth monitoring” "Growth monitoring practice" OR GMP) AND ("Less than two years" OR “Under two years” OR Infant OR Infants OR Childs OR Children “0–23 months”) AND (Ethiopia OR "Ethiopia Federal Democratic Republic" OR EFDR))	42	30/01/2024
**From other grey literature source**			33	30/01/2024

### Study selection

**Fig 1:**
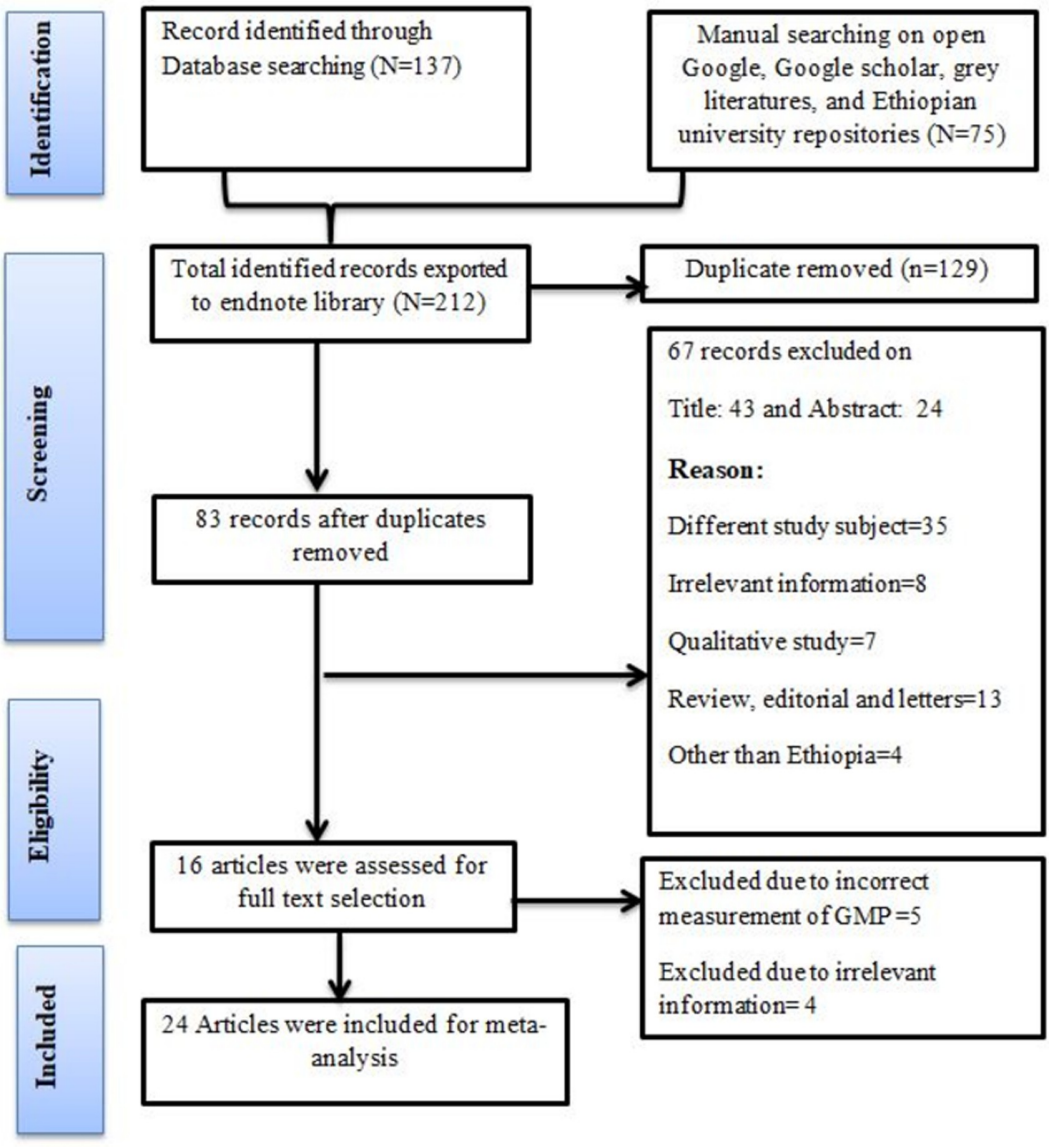
The PRISMA flow chart for the selection of studies for systematic review and meta-analysis on growth monitoring and promotion service utilization in Ethiopia.

All the searched 212 articles were exported to Endnote version 9 to identify the duplicate studies, during which 129 studies were removed due to duplication. Three reviewers (MBS, WMT, and HG) screened the title, abstract and full text selection. The disagreement between the independent reviewers was handled based on selection criteria. Sixty seven (67) studies were removed during title and abstract selection. Sixteen (16) articles were passed for full text assessment of eligibility, and nine of them were excluded from analysis because the study participants and outcome variables were not the same as our review objectives and qualitative studies. Finally, seven (7) studies were included within the prevalence and/or associated factor estimation of growth monitoring and promotion service utilization (**Fig 1**).

### Data extraction

The data were extracted by two reviewers’ (MBS and HG) using a structured Microsoft Excel spreadsheet data extraction form. When variations in the extracted data between the reviewers were noticed, the phase was repeated. If discrepancies between data extractors continued, a third reviewer (WMT) was involved. The name of the author, region, year of publication, study setting (population-based versus institutional-based), study design, data collection method, target population, sample size, prevalence of growth monitoring service utilization, and adjusted odds ratio (AOR) with a 95% confidence interval (CI) of the associated factors with GMP service utilization were collected.

### Risk of bias (quality) assessment

The quality of evidence and risk of bias for studies (case control and cross-sectional) were assessed using the Joanna Briggs Institute (JBI) quality appraisal checklist [25, 26] by two independent authors (MBS and WMT). The disagreement was resolved by the interference of a third reviewer (HG).

**Cross-sectional studies** were assessed using the subsequent items: Inclusion criteria; identification of confounders; strategies to handle confounders; description of study subject and setting; objective and standard criteria used; Reliable and valid measurement of exposure; outcome measurement; and appropriate statistical analysis. Low-risk studies (when they scored 50% or above on the quality assessment indicators) were considered for review and meta-analysis. We had six cross-sectional studies, and all got a score above 50% of the quality scale, which is low risk and can be included in the study **(Table 2)**.

**Table 2:** JBI Quality appraisal checklist for all included studies of GMP service utilization among children less than two years in Ethiopia, 2023.

Included studies		JBI Critical Appraisal Checklist for Cross Sectional Studies(Yes, No, Unclear)																																			
		If Yes(>=50%).....low risk(two raters).....1=Yes 2=No 3=Unclear																																			
		Appropriate Inclusion criteria				Description of study subject and setting			Valid and reliable measurement of exposure				Objective and standard criteria used				Identification of confounder				Strategies to handle confounder				Appropriate Outcome measurement					Appropriate statistical analysis				Over all appraisal			
		R1		R2		R1	R2		R 1		R2		R1	R2			R 1		R2		R 1		R2		R 1			R2		R1		R2					
1.Yeshaneh et al		1		1		1	1		1		2		2	2			1		1		1		1		1			1		1		1		0.8125			
2.Tufa et al		1		1		1	1		1		1		1	1			1		1		1		1		1			1		2		2		0.875			
3.Kiros et al		1		1		1	1		1		1		1	1			2		2		2		1		1			1		1		1		0.8125			
4.Girma et al		1		1		1	1		1		1		1	1			2		2		2		1		1			1		1		1		0.8125			
5.Feleke et al		1		2		1	1		1		1		1	1			1		1		1		1		1			1		1		1		0.9375			
6.Endale et al		2		2		1	1		1		1		1	1			1		1		1		1		1			1		1		1		0.875			
JBI Quality appraisal of included case-control study for growth monitoring and promotion service utilization in Ethiopia, 2023																																					
If Yes(> = 50%)…‥low risk(two raters)…‥>1 = Yes 2 = No 3 = Unclear																																					
Included study	Group comparable among presence of disease in case and absence of disease in control				Case and control matched appropriately			Similar criteria used for case and control				Standard exposure measurement				Similar exposure measurement for case and control				Identify confounding factor				Strategies deal with confounding factor stated			Standard outcome measurement for case and control				Long period of interest for exposure			Appropriate statistical analysis			Over all appraisal
	R1		R2		R1		R2	R1		R2		R1			R2	R1		R2		R1		R2		R1		R2	R1		R2		R1		R2		R1	R2	
7.Dagne et al	1		2		1		1	1		1		1			1	1		1		1		1		3		1	1		1		1		1		2	1	0.85

Footnote, R1; Researcher 1, R2; Researcher 2

**Case-control study** was assessed using the subsequent items: standard measurement of exposure; comparable groups; appropriateness of duration for exposure; appropriateness of cases and controls; similarity in measurement of exposure for controls and cases; criteria for defining cases and controls; handling of confounders; strategies to handle confounders; standard assessment of outcomes; and appropriateness of statistical analysis. The study got 50% or above on the quality scale, which was considered low-risk. We had only one case-control study with a quality score of 85%, which was included in the study (**Table 2**).

### Data synthesis and analysis

The current investigation involved the extraction of AOR, proportion of GMP utilization, and all other relevant data, which was entered into a Microsoft Excel spreadsheet, which was subsequently imported into STATA software version 17 for analysis. The data synthesis was done through a descriptive summary of the included studies via tables and figures. If the studies were appropriate for quantitative synthesis, a meta-analysis was conducted. The estimates from the studies (proportions and odds ratios) were pooled using a meta-analysis regression model to obtain an overall summary estimate. For the meta-analysis, we considered estimates of the adjusted odds ratio with the confidence interval (CI) as the measure of association. The overall effect (pooled estimates of the magnitude and the factors) of GMP was estimated using a random effect model and measured by the prevalence rates and odds ratio with a 95% CI. A random effect model was selected for analysis because of heterogeneity due to differences in the study design, sample size, study year, and study regions. To determine the presence of statistical heterogeneity, the I² test was used. The level of heterogeneity among the studies was quantified, and substantial heterogeneity was assumed due to I² value was greater than 75% [27], which was 96.5% in our case. Pooled analysis was conducted using the Laird random-effects model [28]. Subgroup analysis was done by the study setting (region) and year of publication. A sensitivity analysis was used to assess the influence of individual studies on the overall prevalence estimate of the meta-analysis. Publication bias was checked by the funnel plot and, more objectively, through Egger’s regression test [29]. Prevalence and OR with a 95% CI were used as effect measures.

## Result

The total literature search gave us 212 articles (PubMed = 15, Epistemonikos = 14, Cochrane Library = 25, Science Direct = 20, Embase = 29, Scopus = 34, Google, Google Scholar, and Gray Literature = 75). After the removal of 129 duplicate studies, 83 studies were screened by reviewing their title and abstract, of which 67 were removed. The full text of 16 studies was assessed, and 9 of them were irrelevant. Finally, 7 studies were considered suitable for inclusion in this meta-analysis.

Among the 7 studies included in this analysis, only one was conducted with an unmatched case-control design [15], and the rest six were conducted with a community-based cross-sectional study design [9, 14, 16–19] with a sample size ranging from 363 [15], to 1042 [19]. The year of publication ranged from 2017 to 2023, two studies were published in 2021 [15, 16], 2022 [14, 18], and 2023 [17, 19] respectively. The remaining one study was published in 2017 [9]. All studies were checked for risk of bias and found to be low-risk. Generally, six studies were published within five years, and only one was published before five years. The seven studies included children aged 0 to 23 months. Correspondingly, the magnitude of growth monitoring and promotion service utilization was determined by pooling findings of 7 [9, 14–19]. All this fining is highly strengthen by **(Table 3)**.

**Table 3:** Characteristics of studies included in the systematic review and meta-analysis of growth monitoring and promotion in Ethiopia, 2023.

Author, Year	Study region	Participants	Study setting	Methods employed	Study design	S/Size	Proportion	Quality status
Yeshaneh et al, 2021	Amhara	0–23 month children	Community based	Structured	Cross-sectional	572	38.9	Low Risk
Tufa et al, 2022	Oromia	0–23 month children	Community based	Semi-structured	Cross-sectional	372	25.2	Low Risk
Kiros et al, 2023	Afar	0–23 month children	Community based	Structured	Cross-sectional	416	15.9	Low Risk
Girma et al, 2023	Oromia	0–23 month child mother	Community based	Semi-structured	Cross-sectional	1042	32.8	Low Risk
Feleke et al, 2017	SNNPR	0–23 month children	Community based	Structured	Cross-sectional	819	16.9	Low Risk
Endale et al, 2022	SNNPR	0–23 month children	Community based	Structured	Cross-sectional	443	32.9	Low Risk
Dagne, et al, 2021	Amhara	0–23 month children	Community based	Structured	case-control	363	27.5	Low Risk

### Meta-analysis

#### Heterogeneity of studies

With Cochrane I^2^ values of 96.00%, there was a significant level of heterogeneity across the included studies for the growth monitoring and promotion study. The random effect model was employed to determine the pooled estimates in order to take this significant variability into account.

#### Prevalence of GMP (growth monitoring and promotion service)

**Fig 2:**
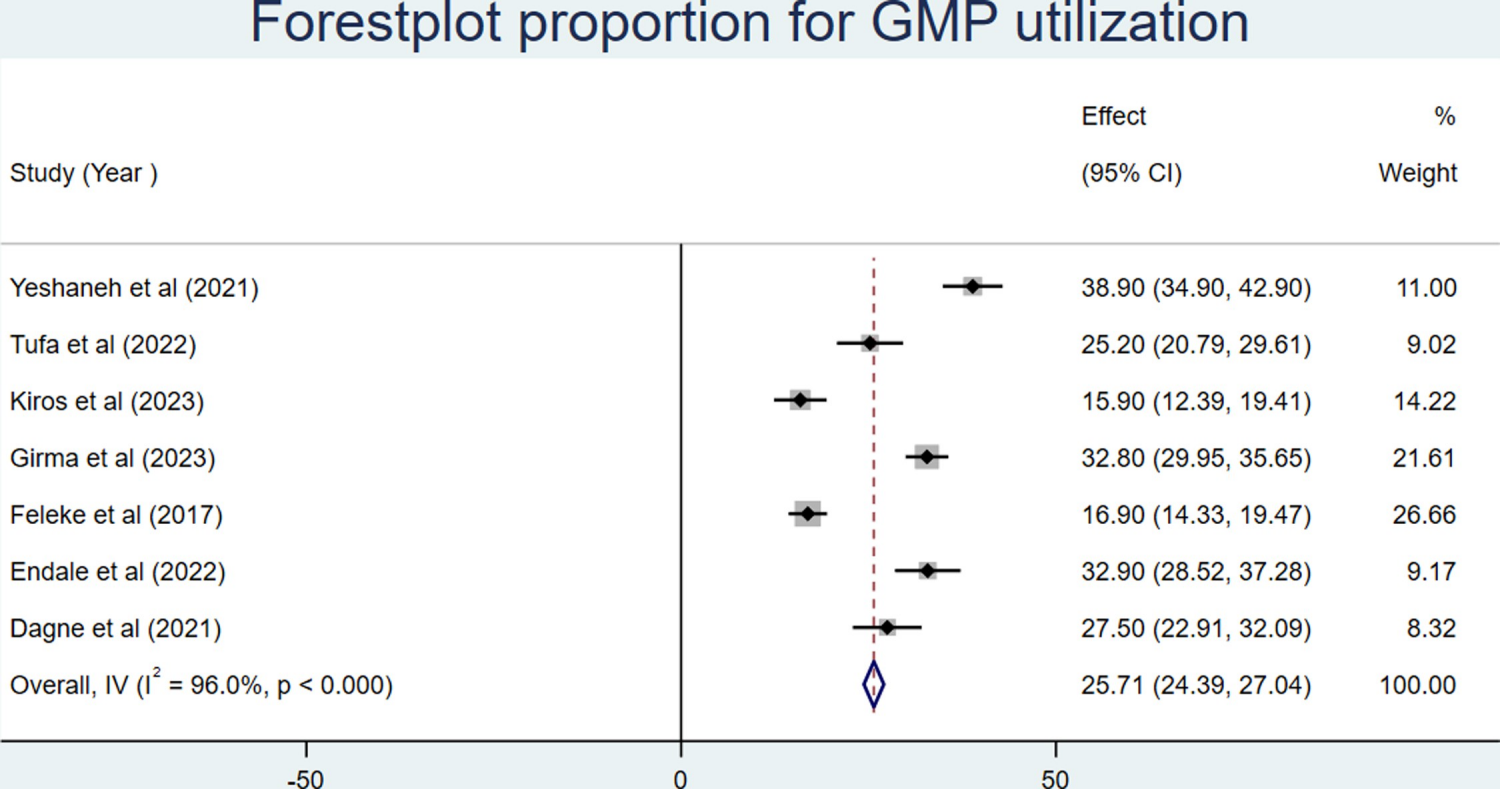
Forest plot of the proportion of growth monitoring and promotion service utilization with corresponding 95% CIs.

The prevalence of growth monitoring and promotion in Ethiopia ranged from 15.9% [17] in Afar to 38.9% [16] in the Amara region. The results of 7 studies indicated that the overall pooled prevalence of growth monitoring and promotion in Ethiopia was 25.71% (95%CI: 24.39, 27.04) (**Fig 2**).

#### Publication bias

**Fig 3:**
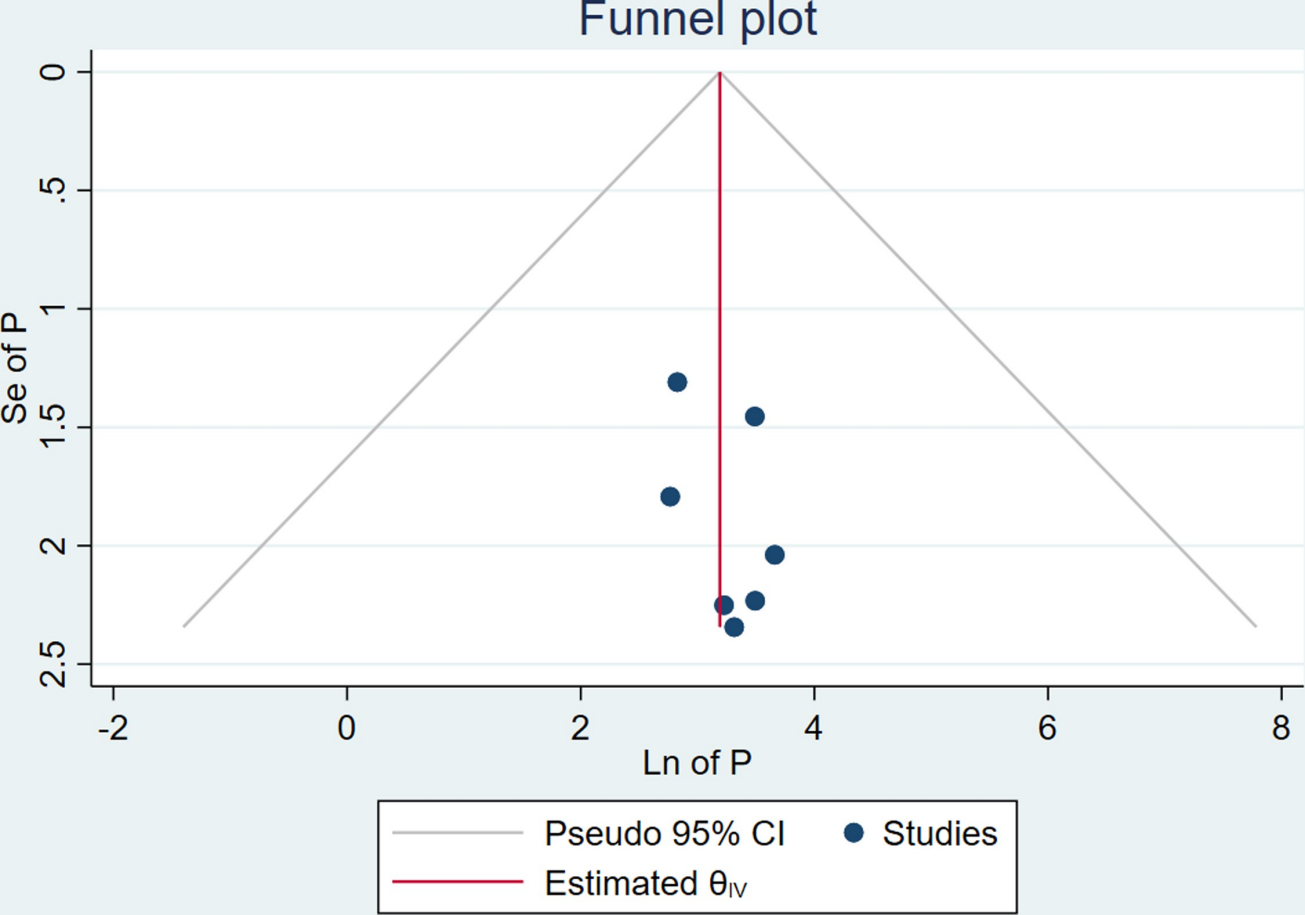
Funnel plot of publication bias for growth monitoring and promotion service utilization among children’s less than two years in Ethiopia; and insignificant Egger’s regression test p-value was 0.829, which indicated the absence of publication bias.

The publication bias could be assessed by using either a funnel plot or an Eggers regression test. For this review, there was no evidence of publication bias as depicted by the symmetrical funnel plot (**Fig 3**).

#### Subgroup analysis

**Fig 4:**
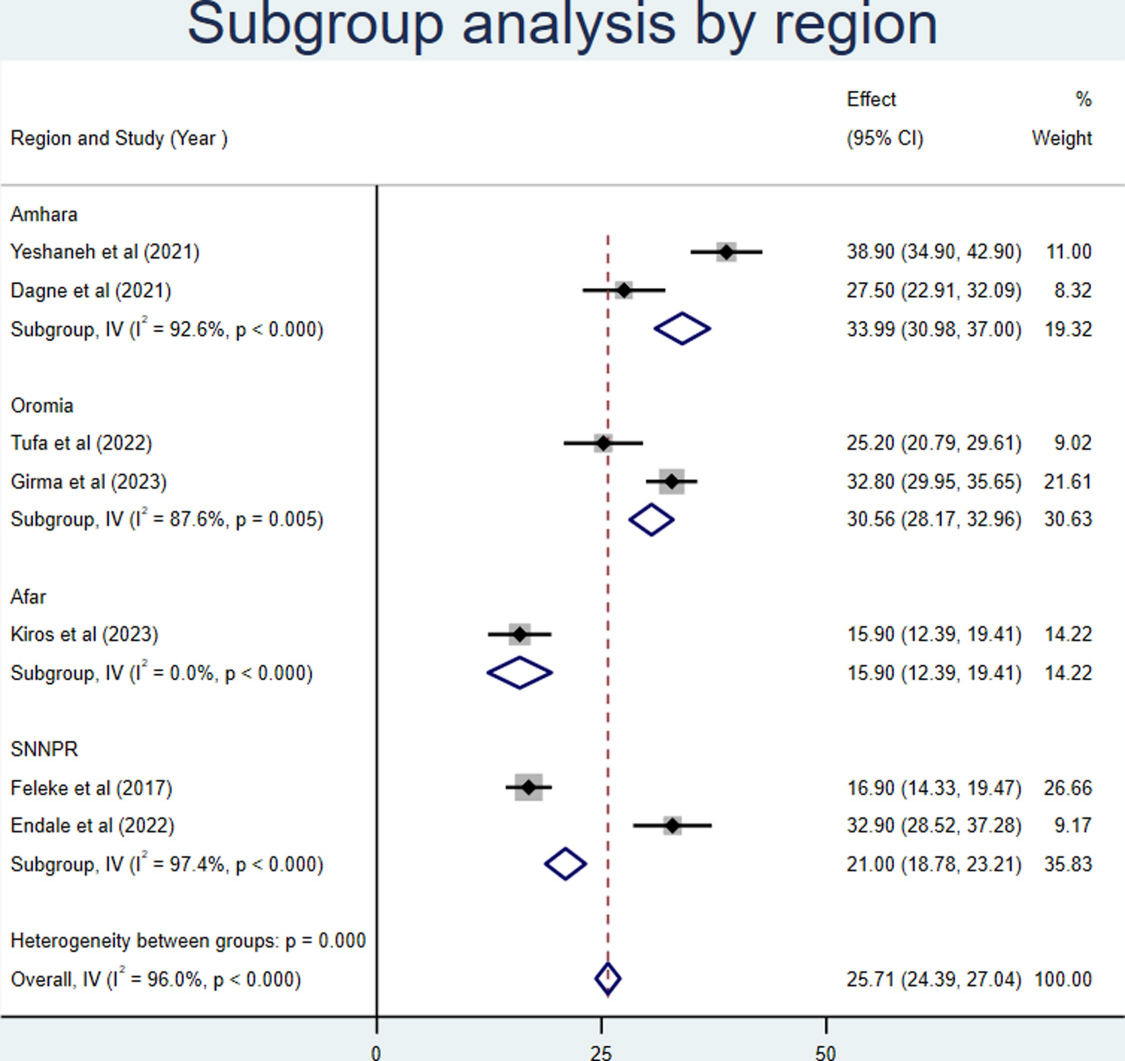
Forest plot of subgroup analysis by region for growth monitoring and promotion service utilization in Ethiopia, 2023.

The subgroup analysis was conducted based on the characteristics of the region and the year of publication. The findings revealed that the utilization of growth monitoring and promotion was found to be 33.99%, 30.56%, 15.90%, and 21% in the Amhara, Oromia, Afar, and SNNPR studies, respectively (**Fig 4**).

**Fig 5:**
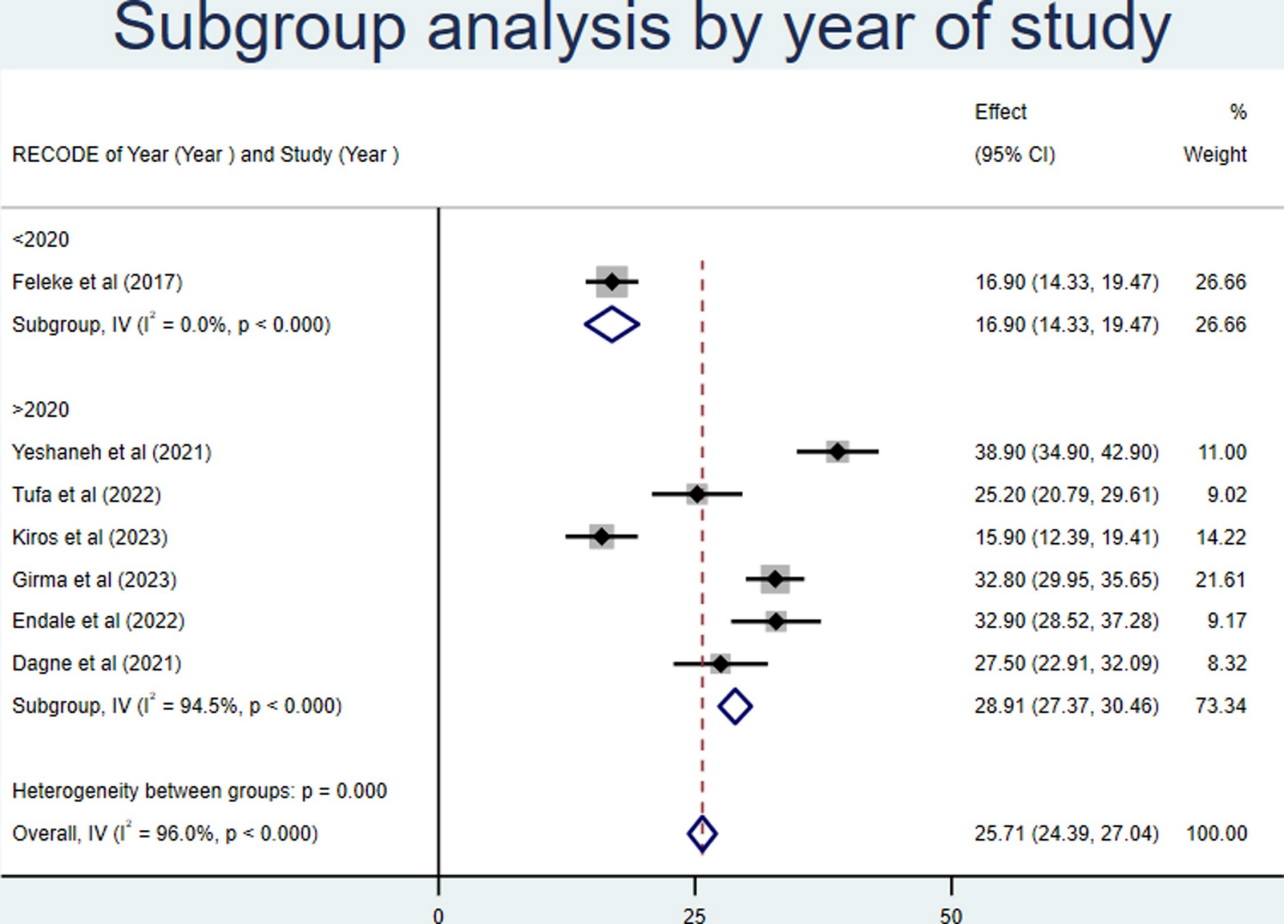
Forest plot of subgroup analysis by year of publication for growth monitoring and promotion service utilization in Ethiopia, 2023.

On the other hand, the subgroup analysis was also conducted for the proportion of growth monitoring and promotion service utilization by year of publication, considering studies published before 2020, and after 2020. We found that the proportion was 16.9% and 28.91% before and after 2020, respectively (**Fig 5**).

#### Sensitivity analysis

**Fig 6:**
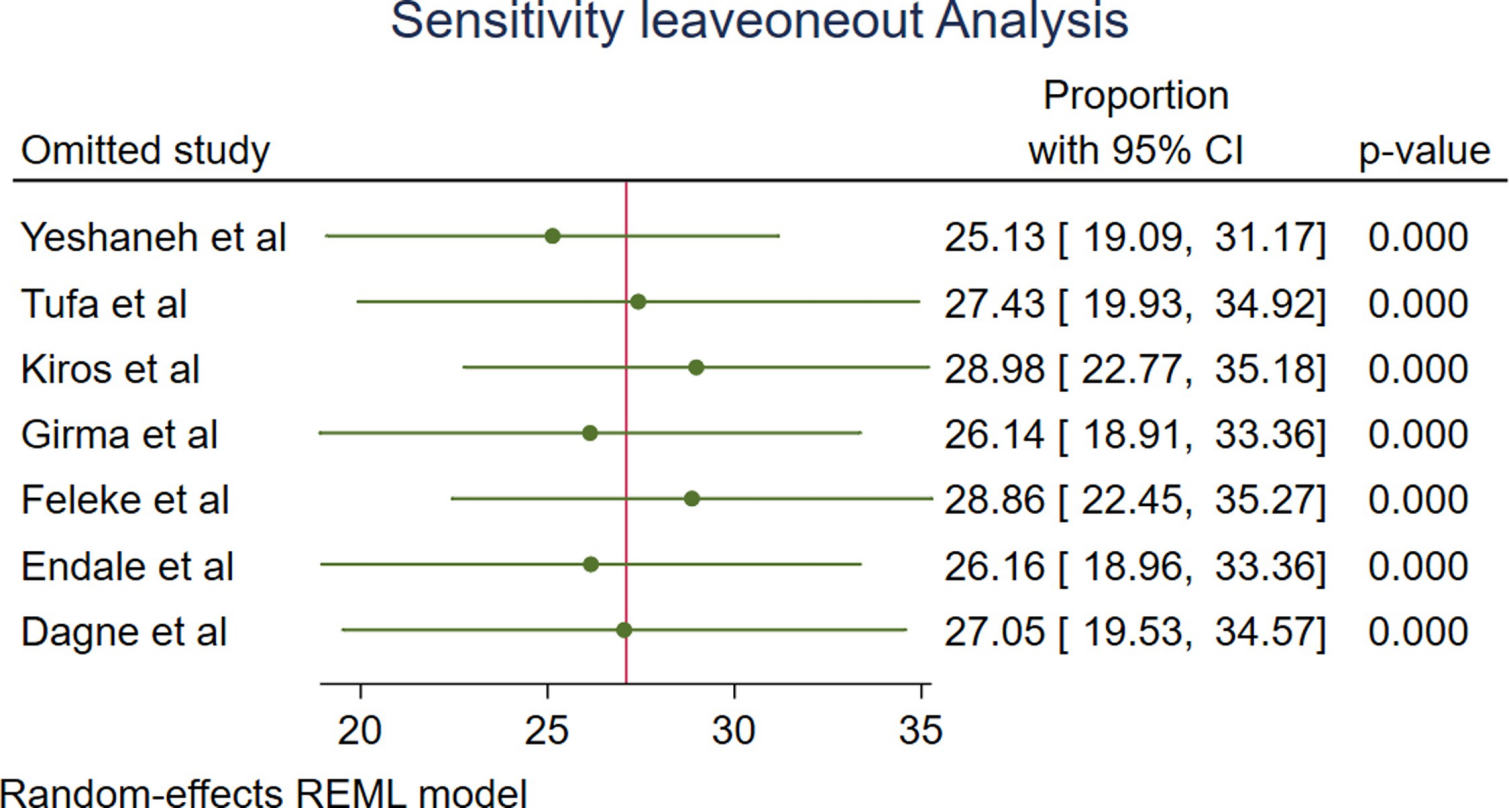
The sensitivity analysis showed the pooled proportion when the studies omitted step by step.

A leave-one-out sensitivity analysis was employed to investigate the influence of each study on the overall estimate. As a result, the pooled estimates of growth monitoring and promotion among 0–23 month-old in Ethiopia were steady and reliable when analyzed by omitting one study at a time (**Fig 6**).

#### Factors associated with growth monitoring and promotion service

Data about ten different variables (age group of the child’s, maternal education, paternal education, ANC follow up, PNC follow-up, family health card, knowledge, attitude, nearby distance to health facility, and counseling) were extracted in pre piloted Excel sheet and analyzed separately. As a result, the pooled effect size of six variables (ANC, PNC, counseling, family health card, maternal education, paternal education, and knowledge) showed a significant association with growth monitoring and promotion service utilization.

Accordingly, three studies were included to assess the association between ANC follow-up and GMP service utilization [9, 14, 15]. Those with ANC follow-up were 2.11times more likely to use GMP as compared to those who did not have ANC follow-up (AOR = 2.11; 95% CI: 1.47, 2.76). Five studies were included to assess the association between PNC and GMP [9, 14, 15, 17, 19]. Mothers who had PNC follow up were 1.96 times more likely to use the GMP service as compared to those who did not have PNC follow-up (AOR = 1.96; 95% CI: 1.44, 2.49). The pooled effect of three studies [15, 18, 19] showed that counseling on GMP was 2.88 times more likely to use GMP service utilization while compared to non-counseling mothers (AOR = 2.88; 95% CI: 2.09, 3.68). The combined effect of three studies [15, 16, 18] revealed that having primary and above maternal education was 2.89 times as likely to utilize GMP as compared to having no formal education (AOR = 2.89; 95% CI: 1.66, 4.13). The findings of three studies [9, 15, 17] showed that those with primary and above paternal education were 3.78 times more likely to use GMP services as compared to those with no formal paternal education (AOR = 3.78; 95% CI: 2.25, 5.32). Similarly the three studies [9, 14, 19] pooled the effect of mothers who had used family health card as 2.31 (95% CI: 1.67, 2.96) times more likely to use growth monitoring and promotion service utilization than their counterparts. The three pooled estimate studies [15, 16, 18] found that mother who had knowledge of GMP were 2.9 times more likely to utilize GMP services as compared to those who had no knowledge (AOR = 2.90; 95%CI: 1.72, 4.07) (**Table 4**).

**Table 4:** Summary estimate of AOR for significant factors associated with growth monitoring and promotion service among 0–23 moth children in Ethiopia based on random effect.

Factors	Number of studies	Effect size	95% Confidence interval	Heterogeneity chi-square (I2 (%), p-value)
Have ANC follow-up	3	2.11	(1.47, 2.76)	0, 0.933
Have PNC follow-up	5	1.96	(1.44, 2.49)	0, 0.070
Counseling (Yes)	3	2.88	(2.09, 3.68)	0, 0.673
Maternal education (primary and above)	3	2.89	(1.66, 4.13)	0, 0.156
Paternal education(primary and above)	3	3.78	(2.25, 5.32)	0, 0.527
Use family health card	3	2.31	(1.67, 2.96)	0, 0.755
Knowledge on GMP	3	2.90	(1.72, 4.07)	0, 0.737

## Discussion

One of the strategic goals aimed at enhancing the nutritional status of children under two years old was to encourage the use of GMP. Growth monitoring services are required until the child is two years old in order to break the intergenerational cycle of malnutrition and produce healthy and well-nourished children.

This analysis comprised seven articles with 4027 participants across four Ethiopian regions. This meta-analysis estimated the national prevalence of growth monitoring and promotion service utilization. We discovered that the proportion of children using growth monitoring and promotion services varied from 15.9% [17] in Afar to 38.9% [16] in the Amara region. Accordingly, the national pooled proportion of growth monitoring and promotion service utilization was 25.71% (95%CI: 24.39, 27.04). This result finding is in line with studies conducted in Ghana 26.5% [30] and Nyanza province of Kenya 25% [31]. Whereas, the pooled proportion of GMP service utilization was less than a study conducted in Accra 64% [32], Rwanda 79% [33], Afghanistan 87% [34], South Africa 70% [35], and Uganda 59% [36]. This discrepancy might be the result of variation in study time, study population, study setting, and socio-demographic status of participants. When we came to Kenya specifically, the growth monitoring and practice study was implemented for under five populations, whereas in our setup, the pooled prevalence of GMP in Ethiopia was implemented among 0-23-month-old children. Most of the earlier studies were institution-based, and the study participants were those who came for routine health delivery services. This may increase the chance for the utilization of growth GMP services. Furthermore, the shortage of access to basic health care services and other obstacles that people in developing nations like Ethiopia experience increase their risk of malnutrition and may reduce their use of GMP services.

The present study also identified factors associated with growth monitoring and promotion service utilization. Antenatal and postnatal follow up care services, the use of a family health card, maternal and paternal education, and maternal knowledge of GMP were found to be significant determinants of GMP service utilization. Accordingly, ANC and PNC follow-up were 2.11 and 1.96 times more likely to use the GMP service, respectively, as compared to their counterparts. This association was also reported by previous studies conducted in Kenya [37] and Benin [38]. This congruent finding might be due to the fact that, as part of the ANC and PNC services, child nutrition and health counseling, including a child’s feeding service, were provided during their follow-up time. Therefore, they might have an opportunity to obtain counseling for the growth and development of their children. Moreover, following delivery, the mothers are going to be pleased to take part in the GMP session; dietary guidance is given throughout prenatal care; and most mothers are aware of the assistance they got from the health facilities. Utilization of family health cards was another predictor of GMP services in this study. Those mothers using family health cards were 2.31 times more likely to use GMP services than those who did not. Perhaps family health card utilization encourages mothers to get growth monitoring services. This study also revealed that primary and higher maternal and paternal education was statistically significant factors for GMP service utilization. Correspondingly, the odds of having primary and above maternal and paternal education were 2.89 and 3.78 times more likely to use the GMP service, respectively. This current finding was consistent with the result reported by Indonesia [39]. Similarly, mothers with good knowledge of GMP were 2.9 times more likely to use GMP services than those with poor knowledge. This study finding is congruent with studies conducted in Ghana [30] and Kenya [37]. This might be the mother who is knowledgeable enough to understand the data on the growth monitoring chart, which encourages the use of GMP [40, 41]. This is because mothers with good knowledge have a better understanding of the negative consequences of not using growth monitoring services, which enhances the GMP utilization. Additionally, maternal awareness of GMP promotes infant and young feeding practices (IYCF) and growth monitoring.

## Strength and limitation of the study

This review has a methodologically rigorous meta-analysis, a reported proportion of GMP, an adjusted odds ratio, subgroup analysis, sensitivity analysis, publication bias, and robust indicators to select sound publications to verify the quality of the research results. However, in this review, 86% of the included studies were cross-sectional, which limited our ability to assess the cause-and-effect relationship. Additionally, we found that heterogeneity is highly concerning, and the interpretation could be with cautious.

## Conclusion and recommendation

This systematic review and meta-analysis indicated that growth monitoring and promotion service utilization remain low in Ethiopia. In this meta-analysis, ANC follow-up, PNC follow-up, counseling on GMP, maternal education, paternal education, family health cards, and maternal knowledge towards GMP were significantly associated with growth monitoring and promotion service utilization. Policymakers and program officers may find the review useful in developing appropriate measures for enhancing growth monitoring and service utilization.

## Supporting information

S1 File Pooled forest plot for all significant associated factors of growth monitoring and promotion service utilization among children less than two years old in Ethiopia, 2023. (DOCX)

S2 File List of all studies identified in the literature search. (XLSX)

S1 Checklist PRISMA 2020 checklist. (DOCX)
