# Supplementary figures and images for: Growth monitoring and promotion service utilization and its associated factors among children less than two years in Ethiopia: A systematic review and meta-analysis
Source: PLoS One. 2024 Nov 19;19(11):e0311531. doi: 10.1371/journal.pone.0311531 (PMC11575828; doi:10.1371/journal.pone.0311531)

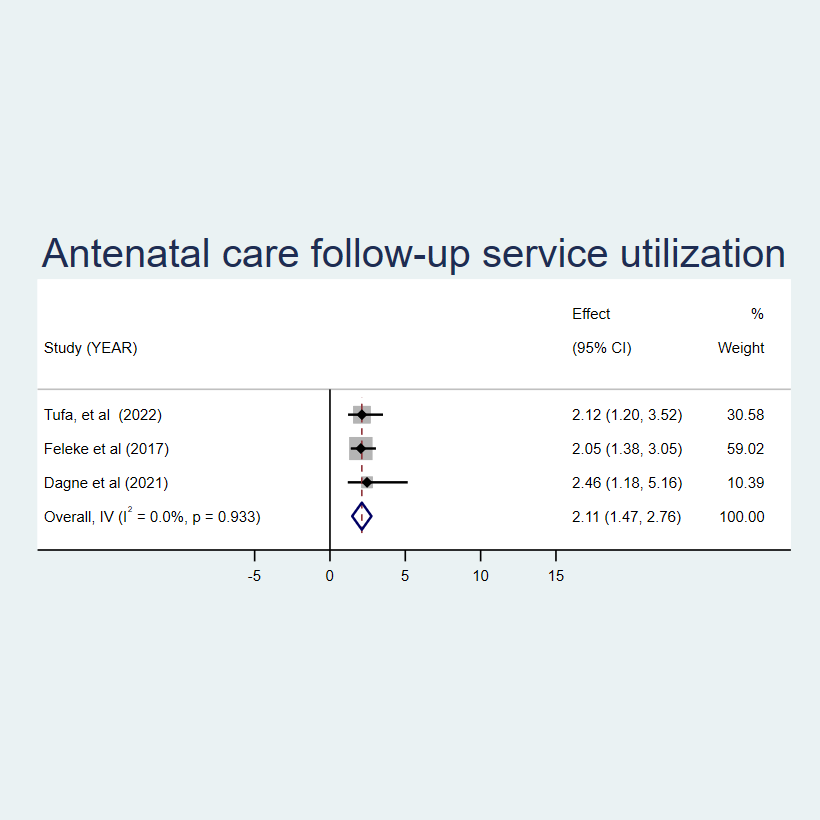


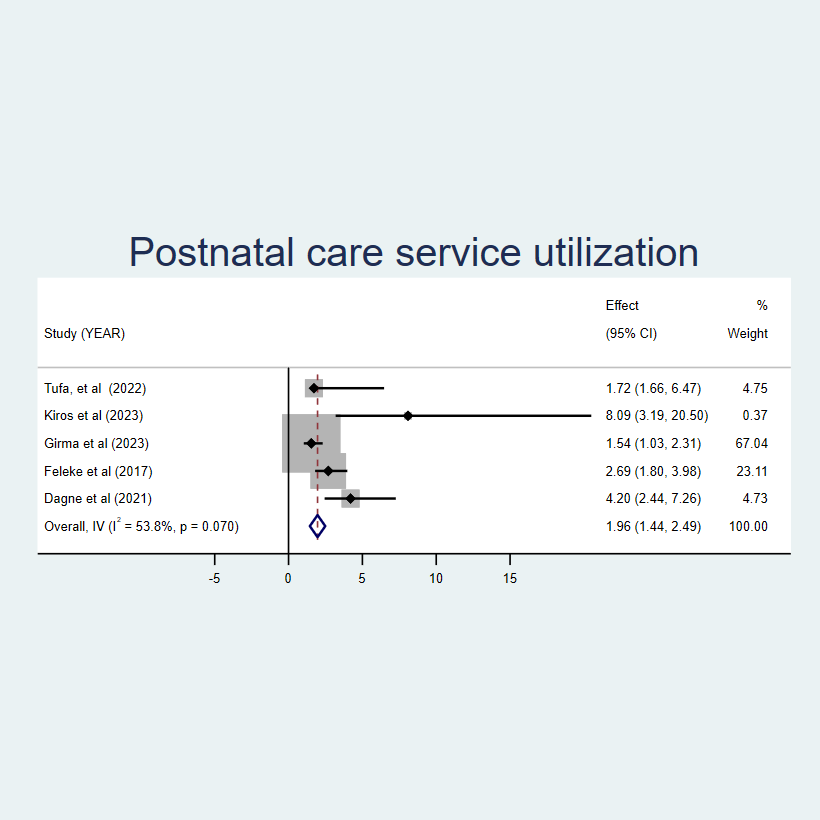


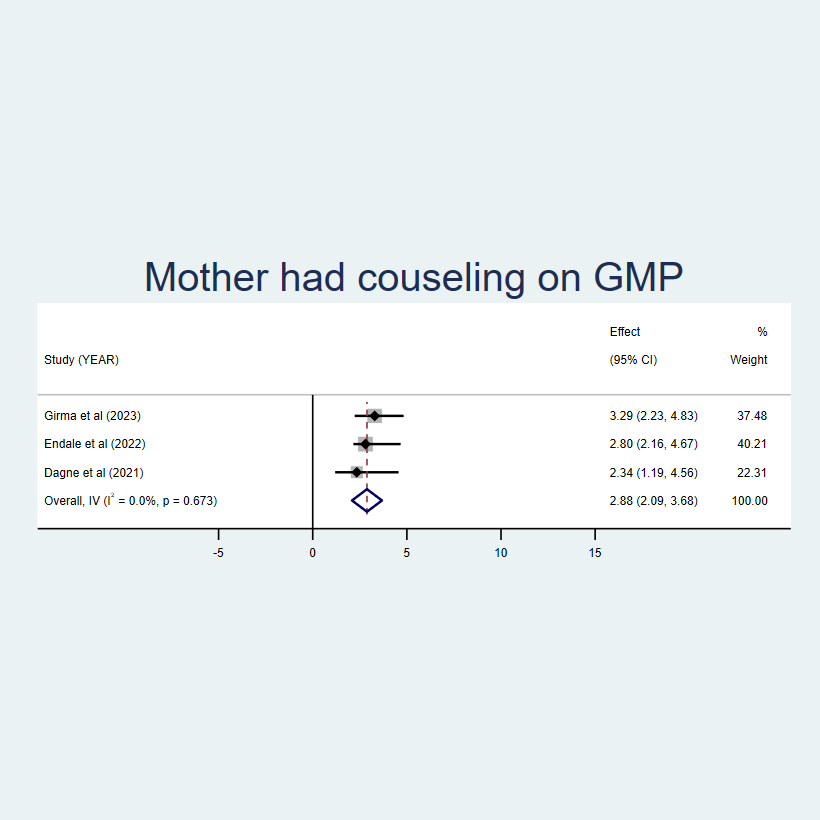


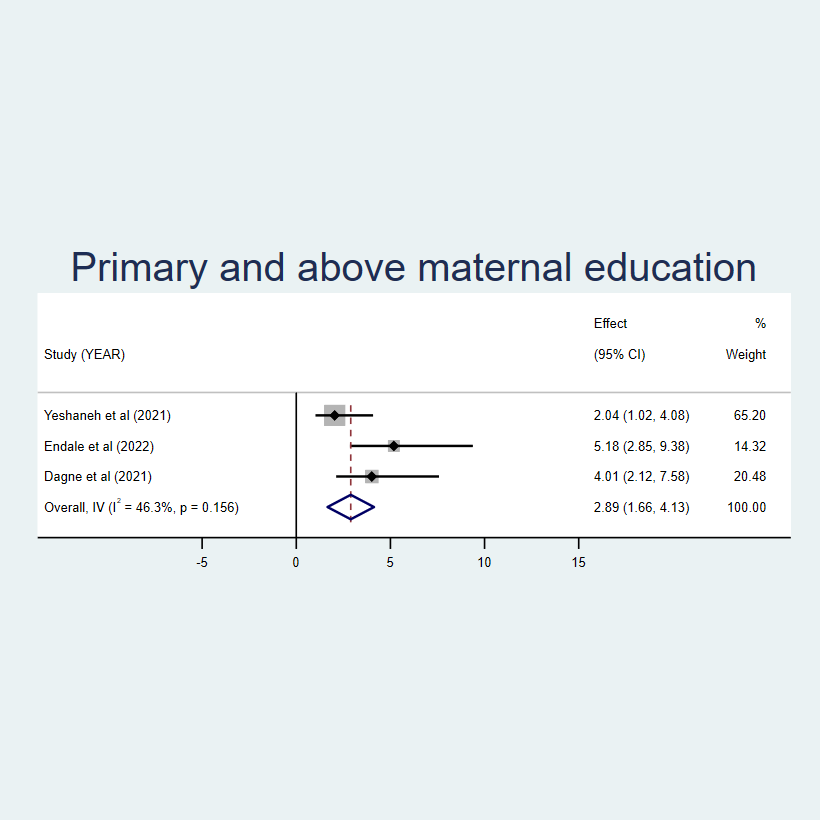


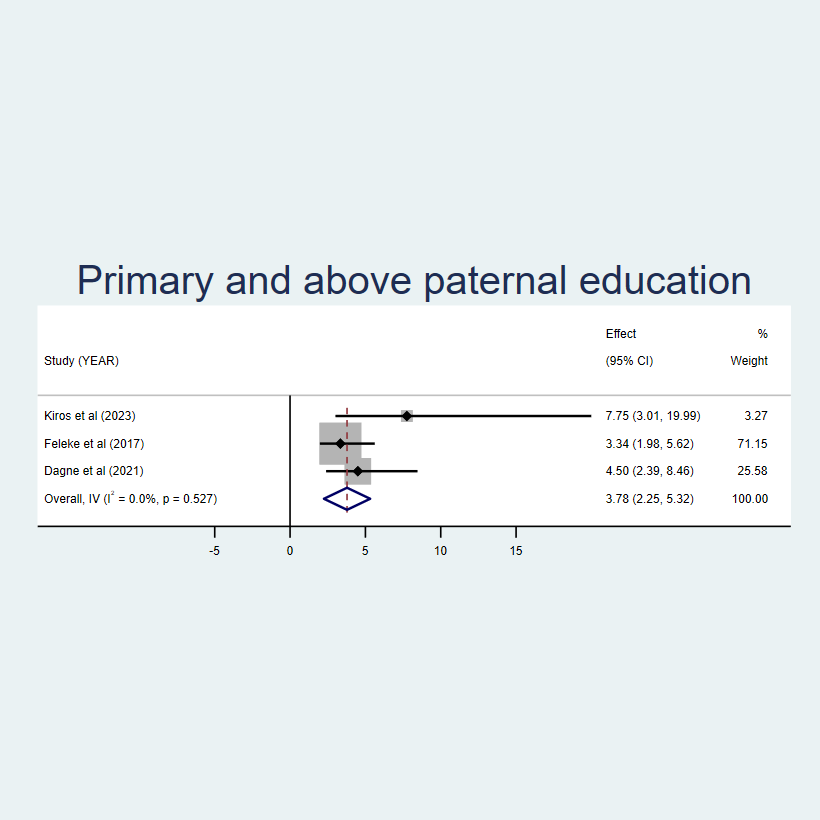


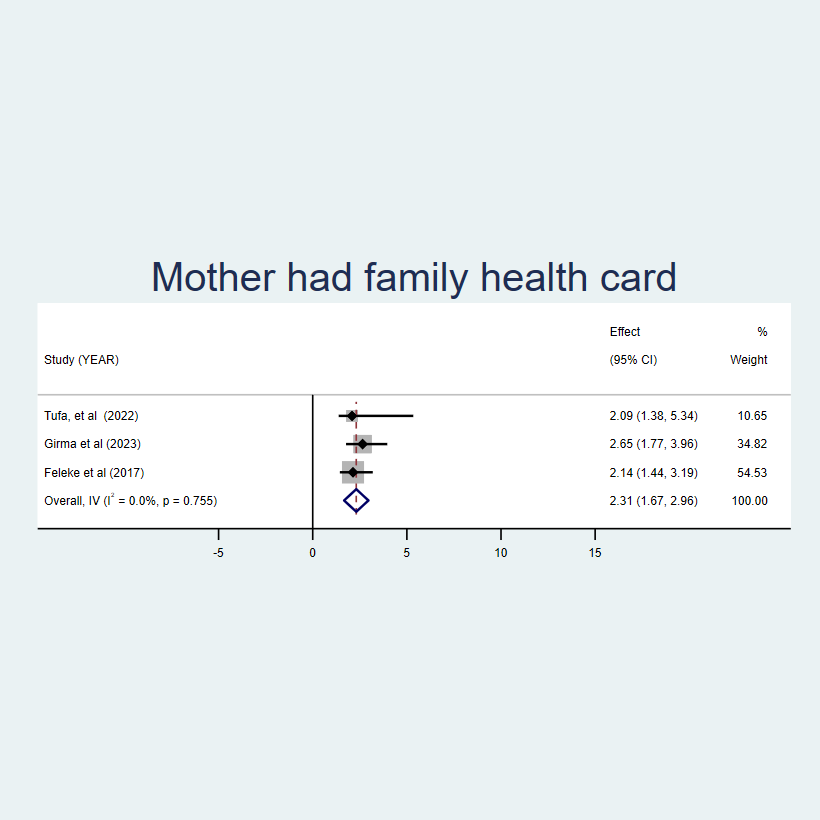


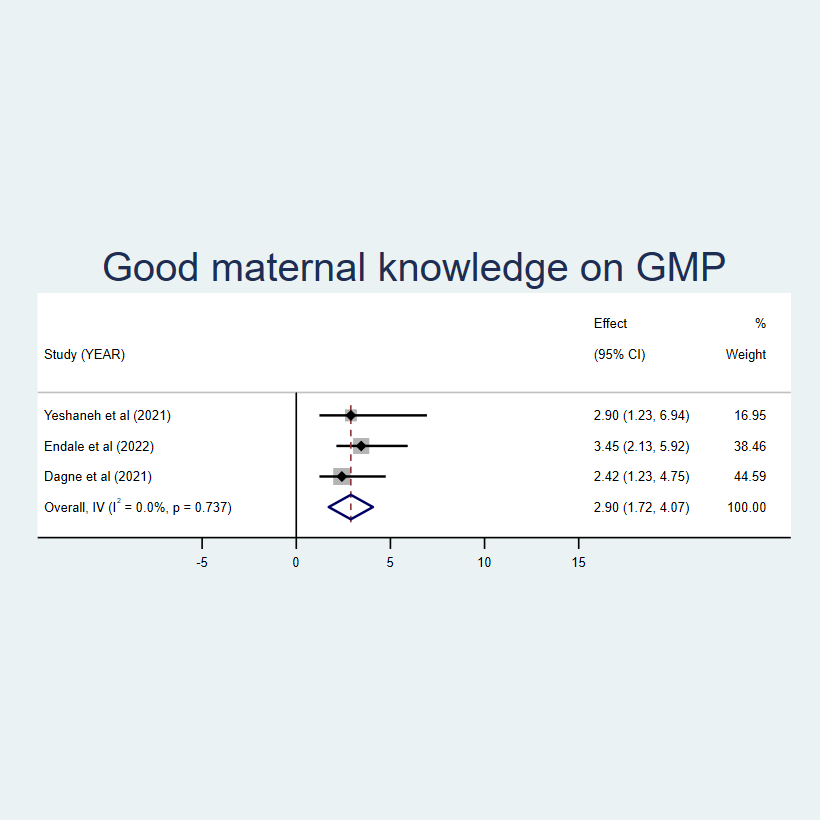

Supplement: S1 File — (DOCX) [file pone.0311531.s001.docx]
